# Relationship between truck driver fatigue and rear-end collision risk

**DOI:** 10.1371/journal.pone.0238738

**Published:** 2020-09-11

**Authors:** Kei Mizuno, Daichi Ojiro, Takeshi Tanaka, Shunsuke Minusa, Hiroyuki Kuriyama, Emi Yamano, Hirohiko Kuratsune, Yasuyoshi Watanabe

**Affiliations:** 1 Laboratory for Pathophysiological and Health Science, RIKEN Center for Biosystems Dynamics Research, Kobe, Hyogo, Japan; 2 RIKEN Compass to Healthy Life Research Complex Program, Kobe, Hyogo, Japan; 3 Osaka City University Center for Health Science Innovation, Osaka, Japan; 4 Department of Medical Science on Fatigue, Osaka City University Graduate School of Medicine, Osaka, Japan; 5 Research & Development Group, Hitachi, Ltd., Tokyo, Japan; 6 Department of Metabolism, Endocrinology, and Molecular Medicine, Osaka City University Graduate School of Medicine, Osaka, Japan; 7 FMCC Co. Ltd., Osaka, Japan; Tongii University, CHINA

## Abstract

The fatigue of truck, bus, and taxi drivers has been a causal trigger for road accidents. However, the relationship between collision risk and the extent of objective fatigue has yet to be confirmed. In this study, we aimed to identify the relationship between autonomic nerve function as an objective parameter of fatigue and the extent of rear-end collision risk, which includes not only objectively risky events but also situations in which truck drivers require safety guidance from safety transport managers. Data of 33 truck driver participants (2 females, 31 males, 46.0 ± 9.1 years old, min–max: 24–65 years old) were analyzed. Drive recorder and automotive sensor data were collected over an eight-month period, and the autonomic nerve function during resting state in drivers was evaluated daily, pre- and post-shift, using pulse waves and electrocardiographic waveform measurement. The rear-end collision risk Index was developed using decision tree analysis of the audiovisual drive recorder data and distance data from the front automotive sensors. The rear-end collision risk index of shift-day was positively correlated with the sympathetic nerve activity index of post-shift condition on the previous day. This suggests that fatigue-related sympathetic nerve overactivity of post-shift condition increases the rear-end collision risk in the following day. Measures, such as actively seeking rest and undertaking fatigue recovery according to the degree of sympathetic nerve activity of post-shift condition, are necessary in order to prevent truck drivers’ rear-end collisions.

## Introduction

In recent years, Japan has witnessed the emergence of a labor environment characterized with long shift hours and driver shortage caused by aging, negative health impacts of driver fatigue [1, 2], and annual increases in accidents related to driver health [3]. Particularly in the case of serious accidents, collisions caused by driver fatigue have become a social issue, with “falling asleep at the wheel” and “careless driving” reported as major causal factors [3]. A previous study indicated that fatigue is a significant influencing factor of driver injury severity [4]. To prevent the occurrence of traffic accidents, the Japan Trucking Association (JTA) is actively promoting health management initiatives for driver fatigue [5]. A JTA guideline, for example, recommends confirmation by the management of each driver’s health at the pre-shift roll call, and the measurement of their temperature and blood pressure [6].

However, methods to directly measure driver fatigue on a shift-day are still based on confirmation of the drivers’ subjective fatigue. Consequently, an accurate evaluation of fatigue levels is difficult due to the presentation of fatigue without noticeable subjective symptoms and dishonesty regarding the degree of fatigue. Therefore, a method to quantitatively and objectively measure fatigue prior to driving is necessary to allow drivers and managers to implement the appropriate measures in response to collision risk. Autonomic nerve function evaluation is one method of objective fatigue evaluation [7]. Autonomic nerve function is composed of the sympathetic and parasympathetic nerve functions. Previous studies have reported that sympathetic nerve activity increases due to driving fatigue and parasympathetic nerve activity decreases [8]. Considering these points, measuring the autonomic nerve functions of truck drivers in both pre- and post-shift conditions is necessary to objectively grasp the degree of driver fatigue.

According to domestic commercial vehicle accident statistics, rear-end collisions account for 53% of truck accidents [9]. Many automotive sensors aim to prevent rear-end collision by evaluating collision risk based on calculating the TTC (Time to Collision) from the distance and relative speed between vehicles [10]. However, many truck drivers highlight issues such as the warning sounding even in situations not considered to be dangerous. In response to these challenges, several studies have proposed new indicators {e.g., avoid collisions by rear vehicle deceleration: DCA (deceleration for collision avoidance)} [11]. Rear-end collision risk indicators have not been established yet. Therefore, there is a need to develop collision risk indicators to detect situations that may lead to rear-end collisions, by using the automotive sensor data of trucks.

While developing collision-risk indicators, it is necessary to define the target situations. Previous studies have retrospectively analyzed the relationships between actual crash events and their factors using previously collected datasets [4, 12–15]. In this prospective study, it was needed to accumulate enough data to analyze the relationship between collision risk and objective fatigue, not using previously collected datasets as retrospective study. However, truck driver participants had the sufficient skills to avoid collisions, which caused the difficulty to accumulate the data of actual crash events. Therefore, we defined the target situations of collision risk referring to the studies which involved human annotators manually assessing situations by watching videos [16, 17]. With this method, the target situations of collision risk in our study included not only objectively risky events (e.g., behaviors to avoid a crash) but also situations that require safety guidance provided for truck drivers by safety transport managers (e.g., behaviors of driving close to a car in the front).

In this study, we attempted the indexing of collision risk using data from automotive sensors on trucks to detect situations that could lead to rear-end collisions. Furthermore, we analyzed the relationship between collision risk and objective fatigue level, conducting a correlation analysis of the developed collision risk index and autonomic nerve function of truck drivers in the pre- and post-shift conditions.

## Materials and methods

### Participants

Forty-three truck drivers (4 females and 39 males, mean age ± standard deviation [SD]: 45.7 ± 9.0 years old, min–max: 24–65 years old) without heart disease, from a certain logistics company, participated in our study. This protocol was approved by the Institutional Review Boards (RIKEN, Kobe2 2018–03; Kansai University of Welfare Sciences, 18–13) and the internal review board of the Research & Development group, Hitachi, Ltd, and was conducted in accordance with the Declaration of Helsinki. All participants provided written informed consent prior to enrollment in this study.

### Study design and procedures

Over approximately eight months, both pre- and post-shift conditions, the autonomic nerve function of participants was used as an objective measure of fatigue using the Fatigue Stress Measurement System (VM500, Fatigue Science Laboratory, Osaka, Japan), while subjective feelings of fatigue were measured using the Visual Analogue Scale (VAS). Four participants were excluded from the analysis due to their inability to clearly and accurately record data when undertaking the VAS measurements (i.e., at each measurement attempt the value remained unchanged from the median value). Six participants, whose alarm never sounded while driving, were excluded from the analysis for exhibiting no possibility of collision risk. Accordingly, there were 33 eligible participants (2 females and 31 males, 46.0 ± 9.1 years old, 24–65 years old) in the final analysis.

The VM500 is an autonomic nerve function measurement device that simultaneously measures pulses waves and electrocardiographic waveform. In these tests, heart rate variability in the resting state was recorded for 90-seconds in the pre- and post-shift conditions [18]. Heart rate measurement data with a misdetection of R waves or abnormal RRI in excess of 10% of the total beats was considered unreliable and disregarded from the analysis. Regarding autonomic nerve function measurement, generally, the frequency component of the RRI is related to the activity of the autonomic nerve function. Within the power spectral density (PSD) gained via frequency analysis of a continuous beat interval, the integration of low-frequency (LF) components of the 0.04 to 0.15 Hz frequency band generally represents the degree of activity of the sympathetic nerve. The integration of high-frequency (HF) components of the 0.15 to 0.4 Hz band generally represents the degree of activity of the parasympathetic nerve. The LF/HF ratio represents the balance between sympathetic and parasympathetic nerve activity. Meanwhile, LF and HF are known to be affected by heart rate and aging [19, 20]. Therefore, when measuring circumstances in which heart rate and the participants’ age cannot be controlled, it is preferable to normalize the frequency components by the effects of heart rate and aging being used as an indicator of autonomic nerve activity [21]. However, as the validity of the HF correction value cannot be verified, it was excluded from the analysis indicators [22]. As such, LF/HF and the LF deviation score (*LF*_score_), which is affected by heart rate and aging, were utilized as objective fatigue indicators in this research. The LF deviation score was defined by the following formula:

$${LF}_{score}(LF,\;age)\;=\;\frac{ln(\sqrt{LF}/{RRI}_{average})-\mu_{LF}(age)}{\sigma_{LF}(age)/10}+50$$

where, *μ*_*LF*_(*age*) and *σ*_*LF*_(*age*) are the mean and standard deviation of *age*, respectively, in the LF standard distribution *N*_*LF*;*age*_ that approximates a normal distribution. The *RRI*_average_ is the average interval between beats, and ln (z) is the natural logarithm of z.

### Estimation of rear-end collision risk

In order to quantitatively evaluate the rear-end collision risk, using automotive sensor data, we developed an estimation algorithm regarding situations which could lead to collisions requiring safety management verification (rear-end collision risk situations).

Based on the data collected during participants’ working hours, we made outcomes and input features that represented driver behaviors. Our target situations included not only objectively risky events (e.g., behaviors to avoid a crash) but also situations that require safety guidance for truck drivers to be provided by safety transport managers (e.g., behaviors of driving close to a car in the front). To determine whether situations included a collision risk, we used the video verification method, similar to previous studies [16, 17]. Using the front-facing video footage from drive recorders (ND-DVR30, Pioneer, Tokyo, Japan), situations were visually analyzed by three safety transport managers from a logistics company, who were assigned as Operation Manager by the Minister of Land, Infrastructure, Transport, and Tourism in Japan [23]. Each manager verified the videos recorded from automotive sensors and extracted candidates of collision risk situations. After the extraction, the candidate situations were verified by all managers, and finally, were determined to be either collision risk situations or not. In addition to automotive sensor warning information from the digital tachograph (ITP-WebService, Transtron, Yokohama, Japan) and the anti-collision system (Mobileye570, Mobileye, Israel), data features concerning vehicle behavior during the warning were also utilized as input features in the analysis.

To determine the collision risk index from both automotive sensors and ground data obtained from safety transport managers to assess the necessity of safety confirmation, we generated the collision risk estimation model. Previous studies often used analysis based on Bayesian statistical modeling [4, 12–15]. However, to make a data-driven estimate of the risk in each situation in which actual rear-end crash events did not occur, we adopted non-parametric analysis for collision risk. This is because we mainly intended to conduct exploratory analysis of the relationship between the input features and outcomes without specific model assumptions which requires parametric analysis methods. Previous non-parametric analysis studies used the random forest and other classification methods for analyzing driving behavior [24, 25]. Here, to balance the specific model assumptions with the interpretation of the risk estimated based on vehicle behavior, we specifically selected the decision tree analysis method, which is a simplification of the random forest method. Thus, the collision risk algorithm was generated using decision trees analysis through a data set of the results from visually determined rear-end collision risk situations (response variables) and variables (explanatory variables) produced from the automotive sensor data of the said situations. Details are shown below.

#### Response variable

The collision risk of 285 cases from all the automotive sensor warnings was randomly selected for assessment. Safety transport managers viewed the video, and designated cases with one of two values; 1 for cases with a rear-end collision risk, and 0 for no collision risk. There were 40 cases designated as actual rear-end collision risks.

#### Explanatory variable

Explanatory variables were those indicating vehicle behavior when the warning was sounding, such as the Duration of the warning sound, Maximum/Minimum/Average speed over the duration of the warning, Deviation of speed over the duration, Maximum deceleration per second over the duration (Table 1).

**Table 1:** Example of the automotive sensor index for estimating accident risk.

Explanatory Variable	Example
Duration	13 [sec]
Maximum speed over the duration	87 [km/h]
Minimum speed over the duration	34 [km/h]
Average speed over the duration	56 [km/h]
Deviation of speed over the duration	6 [km/h]
Maximum deceleration per second for the duration	3 [km/h]

Using these response and explanatory variables, we conducted a decision tree analysis using Classification and Regression Trees (CART) in 5-fold cross-validation [26]. In addition, the hyperparameters of the decision tree (e.g. depth of the tree) were selected by the grid search employed.

Using the developed algorithm, the number of risky situations during one day of driving was aggregated and identified as the number of estimated risky situations, *R*. Moreover, since the driving duration for individual truck drivers varied per day, the number of estimated risky situations for one hour (daily collision risk index) *R*_1*hr*_ was calculated by dividing number of risky situations by the driving duration (hours) *WT*.

$$R_{1hr}\;=\;\frac{R}{WT}$$

Furthermore, since there were situations with short work shifts, data for drivers driving for less than three hours in a day were excluded from the analysis.

### Correlation analysis of the fatigue index and accident risk

We conducted a correlation analysis of the objective fatigue indicators, the LF deviation and LF/HF ratio with *R*_1*hr*_. A correlation analysis was also conducted between the VAS score for fatigue and *R*_*1hr*_. These were investigated using the distribution and quantitative correlation coefficient (Pearson’s product-moment correlation coefficient). In addition, taking the *R*_1*hr*_ median value as a standard, groups were classified as a high or low collision risk, and were investigated to determine whether a significant statistical difference in autonomic nerve function existed between the two groups. Since the normal distribution of data was unaffected by the Shapiro-Wilk test, a test of significant difference (Welch’s t-test) with a 5% significance level was performed. All data analyses were performed using Python (3.6) including SciPy (1.0) and scikit-learn(0.18) [27].

## Results

### Accuracy evaluation of estimated collision risk

Examples of the rules from the developed collision risk estimation algorithm are as follows: Duration greater than 79.0 and 5.8 Deviation of speed over the duration less than or equal to 6.5 and a Maximum speed over the duration of greater than 39 (e.g., situations on the highway where inter-vehicle distance warnings and the acceleration and deceleration continued intermittently for a certain period of time). Duration greater than 42.5 and Maximum speed over the duration of less than 39 (e.g., situations where the inter-vehicle distance warning sounded intermittently, such as when in congestion and traveling at slightly lower speeds on local roads).

The accuracy of the algorithm we developed was investigated using 25 cases of separately prepared test data (Table 2).

**Table 2:** Evaluation of the collision risk estimation algorithm.

Predicted	Collision Risk	Non-collision Risk
Actual		
Collision Risk	4	2
Non-collision Risk	2	17

Four cases were estimated as being collision risk situations, and 14 cases were estimated as being non-collision risk situations. Therefore, the developed algorithm estimated collision risk situations with a high degree of accuracy at 84% (21/25 cases). Meanwhile, in the case of automotive sensors, there were a total of 25 cases of warnings sounded and the rate of collision risk estimation was 24% (6/25), indicating low accuracy. Therefore, our collision risk estimation algorithm showed higher accuracy than that of automotive sensors.

### Relationship between the subjective fatigue sensation and accident risk

**Fig 1:**
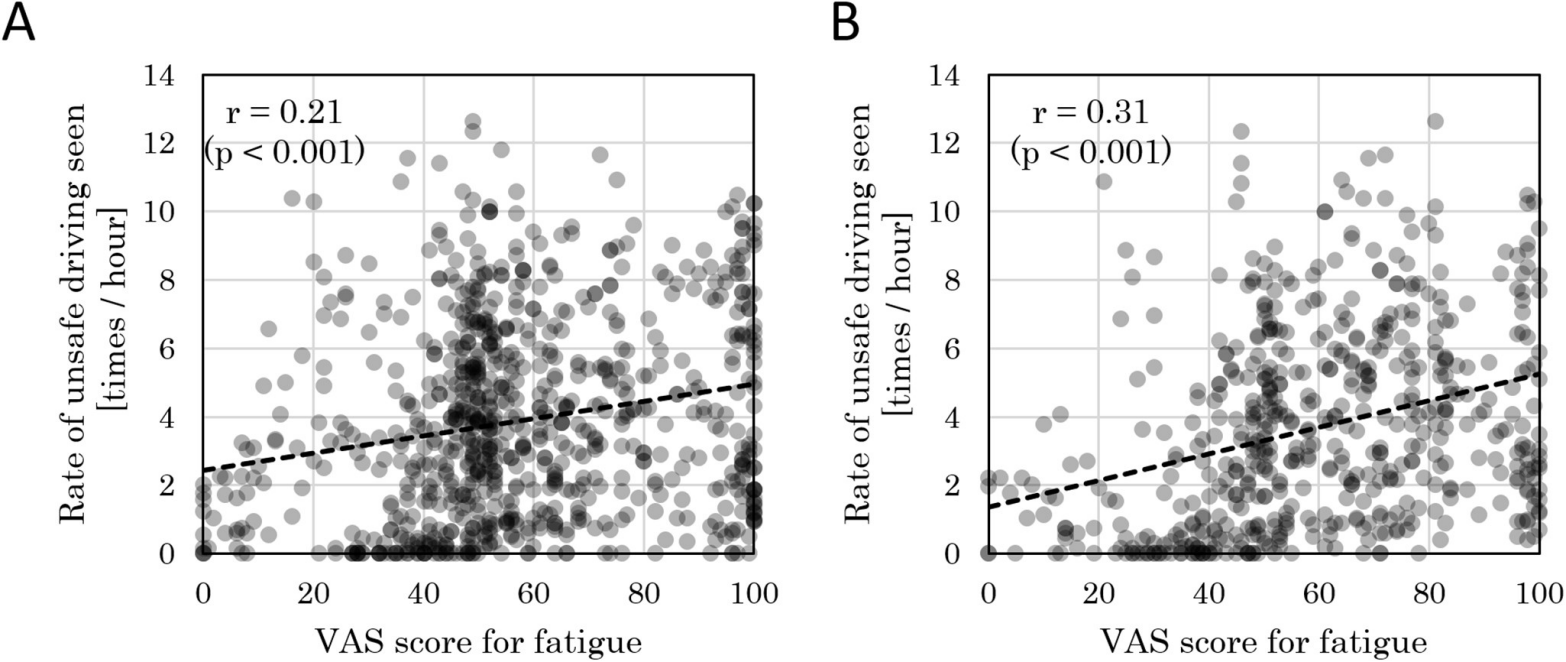
Relationship between the daily collision risk index and VAS for fatigue. (A) Correlation results for visual analogue scale (VAS) score of fatigue in the pre-shift condition, (B) VAS score of fatigue in the post-shift condition, and collision risk judgment frequency (normalized) *R*_*1hr*_. Pearson’s correlation coefficient and the *p*-value are as indicated.

A correlation analysis was conducted using 684 data points, consisting of VAS scores for fatigue in the pre- and post-shift conditions, and the daily collision risk index (*R*_*1hr*_). A positive correlation was found between the VAS score of fatigue in the pre-shift condition and normalized collision risk judgment frequency (Fig 1A). Additionally, a positive correlation was also found between the VAS score of fatigue in the post-shift condition and daily collision risk index (Fig 1B).

### Relationship between objective fatigue measurement and accident risk

**Fig 2:**
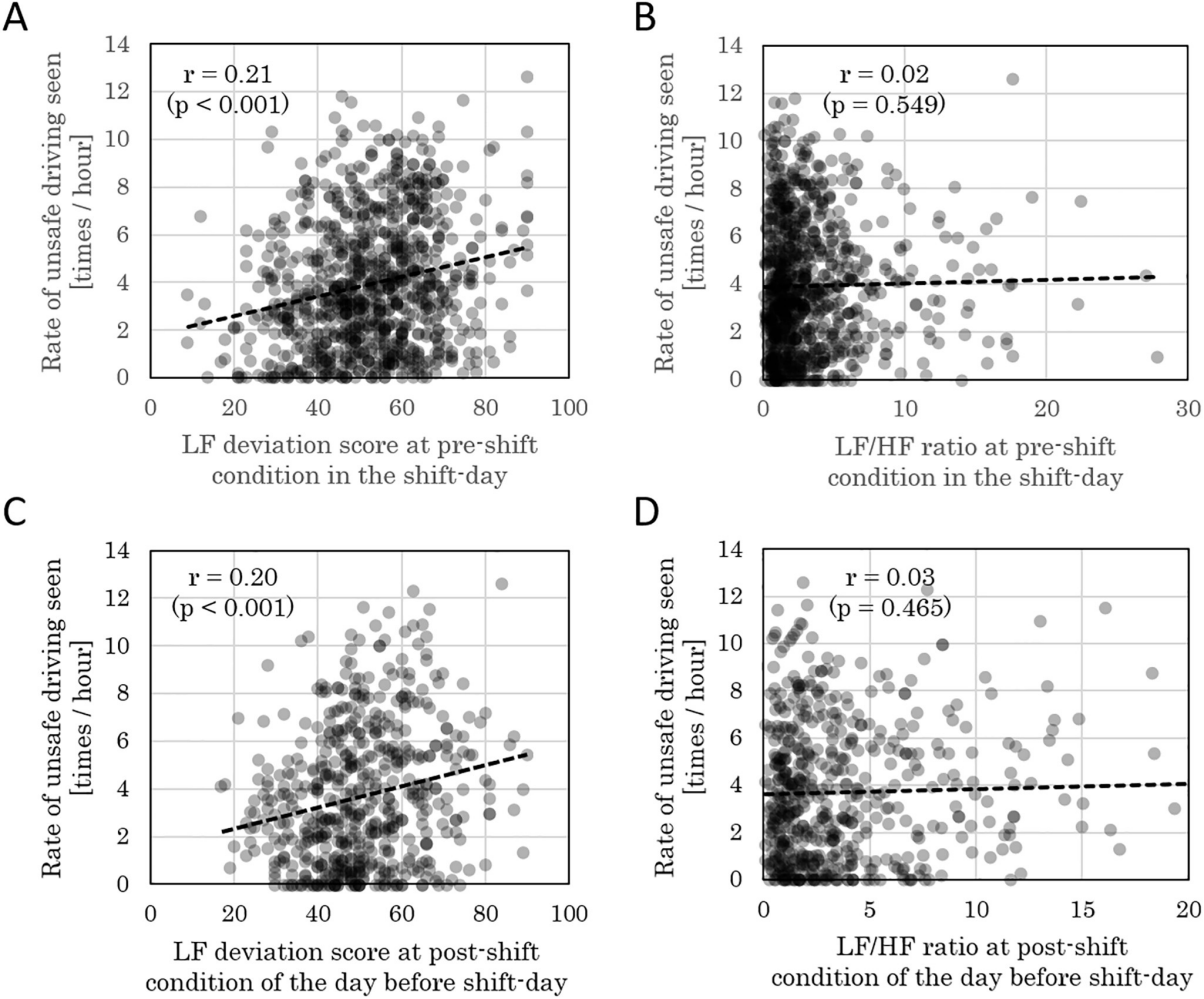
Relationship between collision risk and LF deviation score or LF/HF ratio. Correlation between the shift-day collision risk judgment frequency (daily collision risk index) *R*_*1hr*_ and (A) shift-day pre-shift LF deviation score, (B) LF/HF ratio. Correlation between *R*_1hr_ and (C) LF deviation score of post-shift condition for the day prior to shift-day, (D) LF/HF ratio. Pearson’s correlation coefficient and the *p*-value are as indicated.

**Fig 3:**
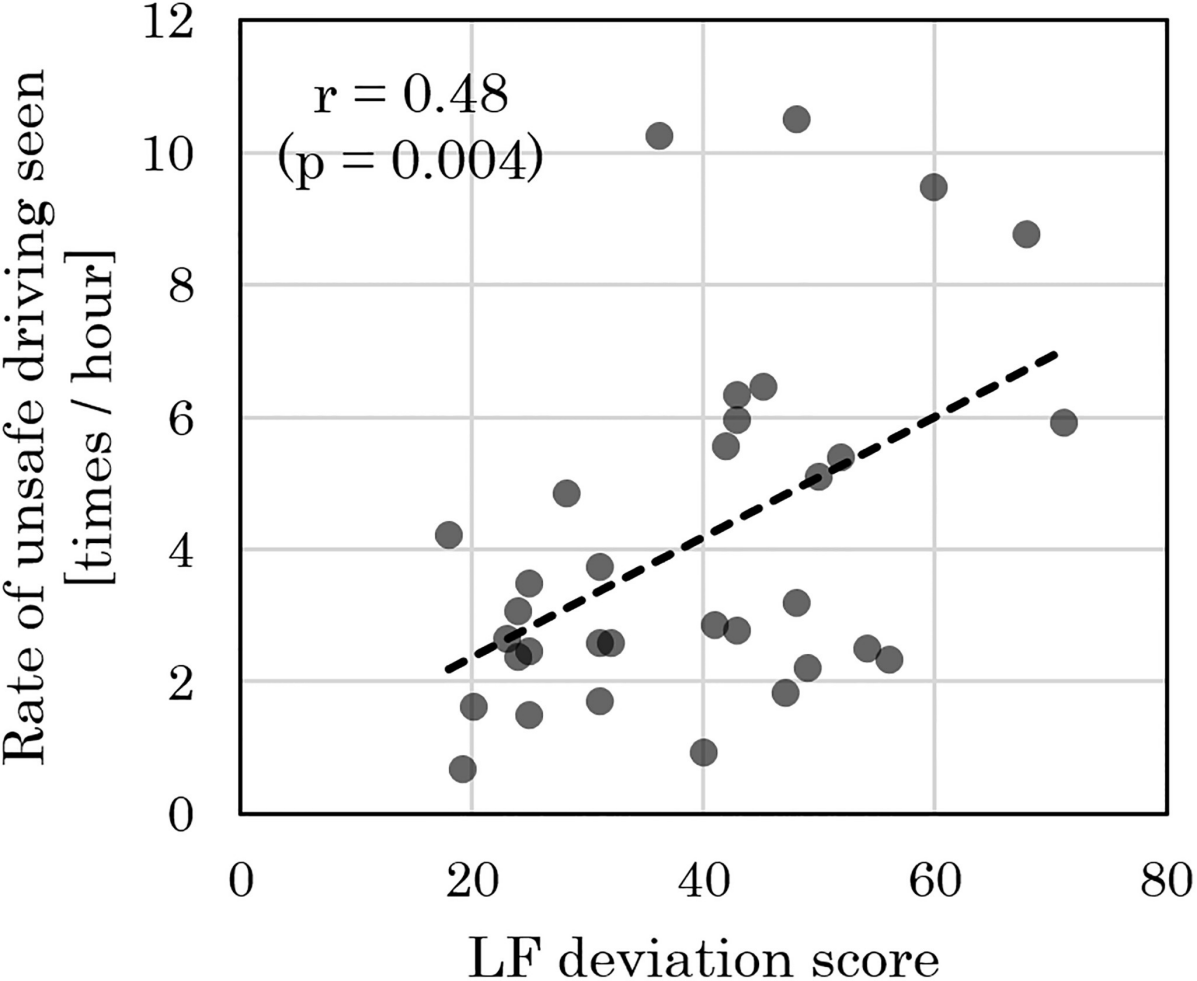
Representative correlation between collision risk and LF deviation score of post-shift condition for the day prior to a shift-day, for a certain driver. Correlation results of a certain driver for the shift-day collision risk judgment frequency (daily collision risk index) *R*_*1hr*_ and post-shift LF deviation score for the day prior to a shift-day. Pearson’s correlation coefficient and the *p*-value are as indicated.

A correlation analysis was conducted using 533 data points, consisting of autonomic nerve function indicators in the pre- and post-shift conditions, and the daily collision risk index (*R*_*1hr*_). While a positive correlation was found for the daily collision risk index in shift-day and LF deviation score of the pre-shift condition in shift-day (Fig 2A), such a correlation was not observed for LF/HF ratio (Fig 2B). Similarly, while a positive correlation was found for the shift-day daily collision risk index and post-shift LF deviation score for the day prior to a shift-day (Fig 2C), such a correlation was not observed for LF/HF ratio (Fig 2D). Fig 3 shows the correlation results of a certain driver for the shift-day daily collision risk index and post-shift LF deviation for a day prior to a shift-day.

**Fig 4:**
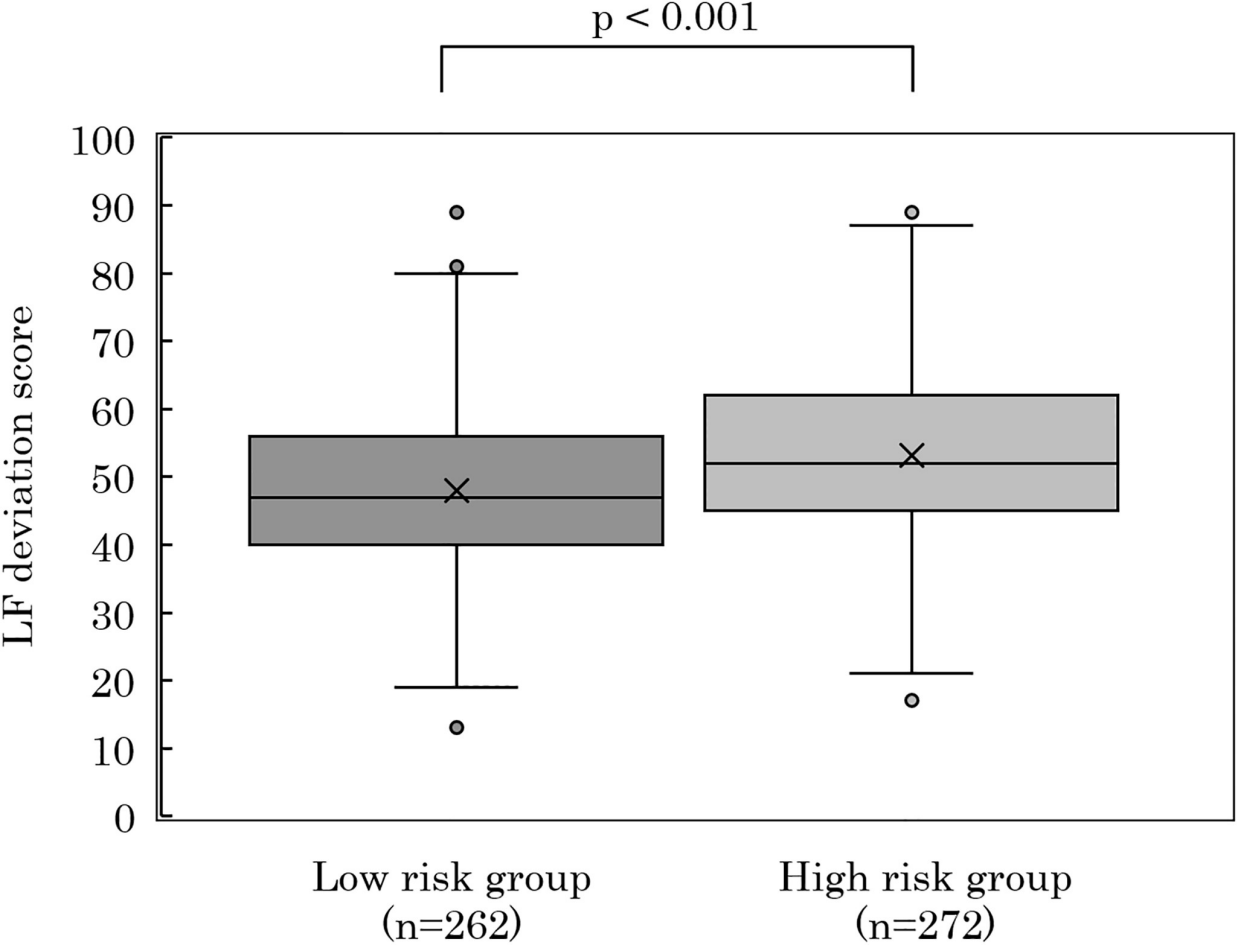
LF deviation scores (post-shift on the previous day) for high- and low-risk groups. Participants divided into high-risk and low-risk groups from the median daily collision risk index *R*_*1hr*_. Comparison of LF deviation scores for high-risk and low-risk groups. *T*-test (Welch’s *t*-test) *p*-value is as indicated.

Participants were divided into high-risk and low-risk groups from the median daily collision risk index (the threshold was set at 3.2 [times/hour]), and a *t*-test of LF deviation (Welch’s *t*-test) was conducted. The high-risk group was found to have significantly higher LF deviation score of the post-shift condition on previous day compared to the low-risk group (Fig 4).

## Discussion

This study revealed that the sympathetic nerve activation, an objective fatigue indicator, of truck drivers increased post-shift condition compared to the previous day, increasing their collision risk during the following day’s shift condition. These results indicate that collision risk can be predicted in advance by evaluating the truck driver’s fatigue in post-shift condition from an autonomic nerve function perspective.

Previous studies that used driving simulators concur with the current results, confirming that increased sympathetic nerve activation results from driving for extended periods of time [8, 28]. Increased sympathetic nerve activation due to fatigue in the post-shift condition involves brain area functions utilized as they drive during a shift condition. Driving is a complex, multitasking activity that involves perception, attention, decision-making, sensory, motor, and higher-level cognitive components [29, 30]. Maintaining a safe driving distance requires continuously evaluating the distance between the cars (i.e., error detection) and choosing between conflict response options (i.e., acceleration and deceleration). Although the dynamics of heart rate variability are used as an index of autonomic nerve activity, the signals of functional magnetic resonance imaging are useful for evaluating neural activities in the brain during a cognitive task (e.g., driving simulation task) [31]. A neuroimaging study of driving simulators revealed that the anterior cingulate cortex plays a crucial role in error monitoring and response selection [32, 33].

As for the autonomic nerve function, a central autonomic net shift that controls the sympathetico-vagal balance is comprised of the orbitofrontal cortex, medial prefrontal cortex, anterior cingulate cortex, insula, amygdala, bed nucleus of the stria terminalis, hypothalamus, periaqueductal gray matter, pons, and medulla oblongata [34, 35]. The anterior cingulate cortex plays a particularly crucial role in the central control of the sympathetico-vagal balance [36]. This indicates that these are the interactions between the activities of car driving dependent regions and autonomic nerve function-associated regions. These results suggest that fatigue, which induces greater activity in the anterior cingulate cortex, a section that corresponds to error monitoring, and response selection during driving results in a sympatho-excitatory response.

Our results illustrate the relationship between high LF deviation score of the pre-shift condition on shift-day and increased collision risk on shift-day. Moreover, it was also shown that high post-shift LF deviation score from the previous day increased collision risk for the following day. Previous day post-shift LF deviation scores were significantly correlated with shift-day pre-shift LF deviation values (r = 0.29, p < 0.001), indicating that the range of variation in LF deviation scores from the previous day post-shift to shift-day pre-shift is minimal. These findings reveal the benefits and significance of allowing drivers time to implement fatigue recovery measures from the previous day post-shift to shift-day pre-shift. Previous research reports that sleep conditions are a related factor for truck driver fatigue [3, 37], with the encouragement of an early bedtime post-shift appearing effective. Furthermore, incorporating solutions proven to improve autonomic nerve function (e.g., listening to healing music [38], performing yoga [39], and hydrogen-rich water intake [40], etc.) into their lifestyle can be useful in fatigue recovery. In addition, having truck drivers learn fatigue management techniques is also considered to be a useful measure to reduce collision risk in the medium to long-term. It was reported that truck drivers who had not completed fatigue management training were also six times more likely to have been involved in a crash [41], suggesting that methods and knowledge of fatigue management may be important in decreasing the fatigue of truck drivers.

In this study, judgment rules were generated by decision tree analysis using the warning duration to develop rear-end collision risk indicators. Consideration of warning sound duration enables judgment of situations, for instance, where a relatively short inter-vehicle distance which is not instantly judged as dangerous is maintained for a period of time (such situations often occur on highways). Since risk estimation in conventional indicators such as the TTC and DCA is based on instantaneous figures at a point in time (e.g., speed, inter-vehicle distance) estimation of the situation is difficult. However, the current algorithm is capable of estimating collision risk situations that are unable of being identified by conventional indicators [10, 11]. Given that the collision risk estimation algorithm was generated from objective data, the automation of rear-end collision risk estimation is possible. It will become possible to easily evaluate collision risk even with long-term vehicle data from large numbers of drivers in the future. In the present study, the collision risk indicator was used in relationship analysis of objective parameters of fatigue, however, the use of the algorithm is not limited to this example. Collision risk is known to be related not only to fatigue, but also to driver skills and driving conditions, such as the weather [37, 42]. The collision risk indicator can also be employed in the analysis of other related factors. Moreover, it could also be employed for driver safety instruction, when algorithm judgment is implemented in day-to-day operations.

Although our results showed that fatigue-related sympathetic nerve overactivity in the post-shift condition increased the rear-end collision risk on the following day, there are some limitations in our study. First, there were only two female participants among the final 33 participants. This gender imbalance may result in biased results, and these effects should be evaluated in future research. In addition, the decision tree analysis, which is a non-parametric method, was adopted to balance the no specific model assumptions and the interpretation of the estimated risk based on automotive behaviors. In future studies, an analysis based on Bayesian statistical modeling, which assumes several mechanisms of collision risk, will enable precise analysis and enhancement of interpretability [4, 12–15].

## Conclusion

In the present study, we aimed to identify the relationship between fatigue, which may cause drivers’ road accidents, and a measure of rear-end collision risk. Our results showed that the rear-end collision risk index on a shift-day was positively correlated with the sympathetic nerve activity index in the post-shift condition on the previous day. This suggests that fatigue-related sympathetic nerve overactivity in the post-shift condition increases the rear-end collision risk on the following day. According to our study, ensuring evaluative measurement of the autonomic nerve function of each driver in the post-shift condition allows for objective monitoring of driver fatigue levels. To improve driver health management by monitoring the level of post-shift sympathetic nerve activation, it is necessary to respond by actively encouraging drivers to rest, and by working toward fatigue recovery. The development of this system will contribute to rear-end collision risk prevention during shifts on the following day. Our findings may provide guidance for further research on the prevention of rear-end collisions and driver health management through objective fatigue evaluation using measurements of the autonomic nerve function.
